# Studies on enzymatic oxidation of 3′,4′-dihydroxy-l-phenylalanine to dopachrome using kinetic isotope effect methods

**DOI:** 10.1007/s10967-013-2867-2

**Published:** 2013-12-10

**Authors:** Wioleta Byszewska, Marianna Kańska

**Affiliations:** Department of Chemistry, University of Warsaw, Pasteur Str. 1, 02-093 Warsaw, Poland

**Keywords:** Deuterium, l-DOPA, Isotope effects, Oxidation, Peroxidase

## Abstract

We report the studies on the mechanism of oxidation of 3′,4′-dihydroxy-l-phenylalanine (l-DOPA) to neurotoxic dopachrome catalyzed by enzyme horseradish peroxidase (EC 1.11.1.7) using the kinetic (KIE), and solvent (SIE), isotope effect methods. For kinetic studies two specifically deuterated isotopomers: [2′,5′,6′-^2^H_3_]-l **-**DOPA was synthesized by the acid catalyzed isotopic exchange between native l-DOPA and heavy water, and [5′-^2^H]-l-DOPA was synthesized in two step reaction. The first step involved acid catalyzed isotopic exchange between l-tyrosine and deuterated water and resulting product [3′,5′-^2^H_2_]-l-tyrosine was hydroxylated by enzyme tyrosinase (EC 1.14.18.1). The values of deuterium KIEs and SIE’s in the enzymatic oxidation of l-DOPA and its isotopomers are determined using non-competitive spectrophotometric method. The measured values were: KIE on *V*_max_ (1.1 and 2.2) and KIE on *V*_max_/*K*_M_ (1.7 and 3.2) for [2′,5′,6′-^2^H_3_]-l-DOPA and [5′-^2^H]-l-DOPA, respectively, while the corresponding values of SIE were: SIE on *V*_max_ (2.1, 2.4, and 2.1) and SIE on *V*_max_/*K*_M_ (1.3. 1.6, and 1.1) for l-DOPA, [2′,5′,6′-^2^H_3_]-l-DOPA, and [5′-^2^H]-l-DOPA, respectively. The size of KIE and SIE, typical for secondary isotope effects indicate that both the solvent and presence of deuterium at the 2′-, 5′, and 6′-positions of l-DOPA has the little impact on the enzymatic oxidation of this compound.

## Introduction

The oxidation of 3′,5′-dihydroxy-l-phenylalanine (l-DOPA) and its derivatives, such as dopamine, epinephrine and norepinephrine, to dopachrome (DC) and other aminochromes, is primary stage of neuromelanins (NM), biosynthesis [1]. The functions of NM in organisms is not quite clear.

However, in the literature, their potential neuroprotective role has been suggested [2]. On the other hand, it seems that these metabolic processes play a neurotoxic action. They might be responsible for the increasing the oxidative stress by formation of several toxic molecules, such as free radicals, reactive quinones or even neurotoxic aminochromes.

**Fig. 1:**
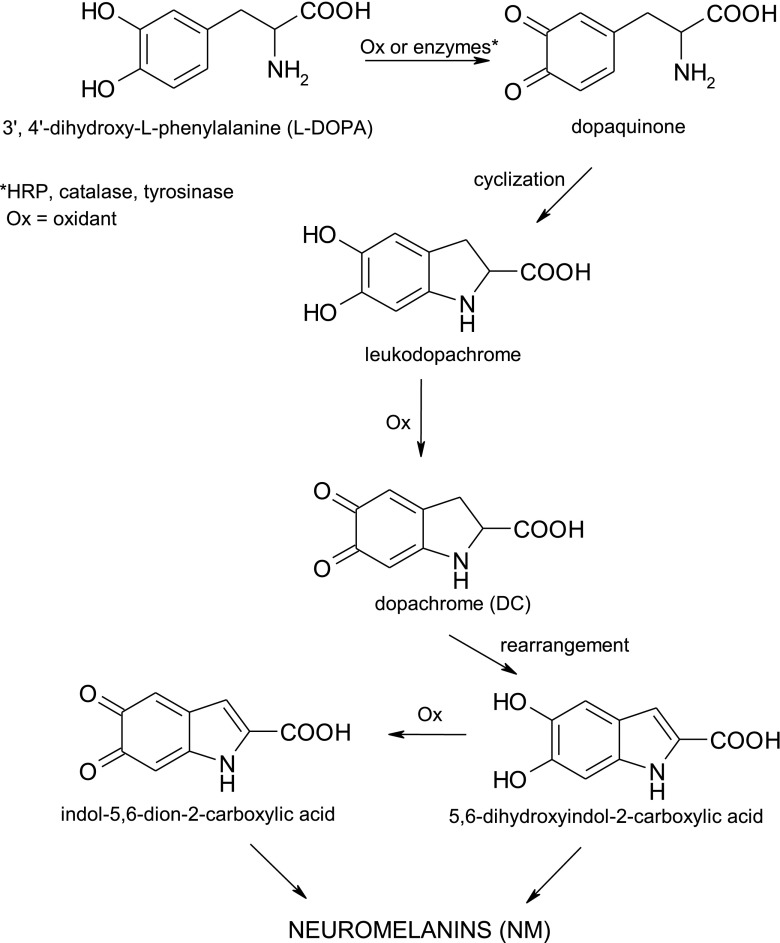
Neuromelanins biosynthesis pathway from l-DOPA

For many years studies on the biosynthesis of NM drawn special interest among researchers, particularly its early stages leading to formation of aminochromes, and several mechanisms were proposed by the kinetic approach to early steps in NM synthesis [3, 4]. However, the knowledge about them remains quite limited. It is more or less established that these oxidation processes take place through four one-electron transfer steps. Through this sequentional way some intermediates are formed to finally be transformed to aminochromes [5–7]. These latter, include dopachrome and its derivatives, may rearrange to indol-5,6-dion derivatives, which can be oxidized to 5,6-dihydroxyindol derivatives and polymerize to NM [2, 8] (Fig. 1). Increased amount of indole derivatives of l-DOPA and other compounds with catechol moiety in nervous system was found to be hazardous to brain function because of inhibition of synaptic transfer of electrical signals similarly to the action of hallucinogens [9]. Therefore, it is supposed that commotion of the normal metabolism of l-DOPA and its derivatives, especially through the production of dopachrome and similar moiety, leads to development of many pathologies, including neurological disorders, such as schizophrenia [9–11]. The reactive oxygen species, forming during the oxidation processes of l-DOPA, can be involved in producing covalent modifications in proteins structure resulted in an impairment of its physiological functions and failures in the normal redox state of cells [2].

The oxidative pathway of compounds with catechol moiety occurring at physiological pH, can be stimulated by both chemical and enzymatic systems [7]. The enzymes such as catalase, tyrosinase or peroxidase systems are capable to oxidizing l-DOPA and its derivatives are quite widespread in nature. As a chemical stimuli of above processes can be listed oxidative agents such as alkalies, metallic cations or free radicals.

Considering the potential high toxicity effects of aminochromes in cells of the nervous system, we focused our attention on the studies on enzymatic transformation of l-DOPA to dopachrome. This issue seems to be crucial for further investigations into causes of schizophrenia and other neurological disorders [12].

For our research we concentrated on the enzyme peroxidase from root of horseradish plant (HRP, EC 1.11.1.7). It belongs to the class of iron-containing oxidases, widely distributed in both plant and animal kingdom. HRP forms a complex with hydrogen peroxide, giving an effective oxidizing system for large variety of substrates, including aromatic and inorganic electron donors [13]. It is now posited that peroxidases play a primary role in the defense organism against oxidative damage of neuronal cells by catching the intracellular H_2_O_2_. However, the mechanisms of these processes have not been clearly established yet.

The aim of our studies is a kinetic analysis of oxidation of l-DOPA to dopachrome, DC catalyzed by enzyme HRP. To investigate this reaction the kinetic, KIE, and solvent, SIE, isotope effect methods were used [14, 15]. Two deuterated isotopomers of l-DOPA needed for this studies: [2′,5′,6′-^2^H_3_]- and [5′-^2^H]-l-DOPA, were obtained using the acid catalyzed isotopic exchange between authentic l-DOPA and heavy water or hydroxylation of [3′,5′-^2^H_2_]-l-tyrosine catalyzed by enzyme tyrosinase (EC 1.14.18.1), respectively. Determination of numerical values of deuterium SIE’s and KIE’s can provide relevant information about mechanism of enzyme-catalyzed oxidation of above amino acid studied.

## Experimental

### Materials

Solutions of 37 % DCl/D_2_O, 30 % NaOD/D_2_O and 83 % D_3_PO_4_/D_2_O needed were obtained from POLATOM, Poland. Deuterated water (99.9 % D) was from Aldrich. Aluminum oxide (aluminum oxide 60 F_254_, neutral, type E) and silica gel 60 F_254_ plates were purchased from Merck. Aluminum oxide for column chromatography was obtained from POCh, Poland. l-tyrosine and l-DOPA were from Aldrich. Enzymes: peroxidase, type II (EC 1.11.1.7) from horseradish, HRP, and tyrosinase (EC 1.14.18.1) from mushroom *Neurospora crasaa*, l-ascorbic acid and other chemicals needed for syntheses and trial experiments were purchased from Sigma.

### Methods

The ^1^H NMR spectra were recorded in D_2_O using TMS as internal standard on a Varian 500 MHz Unity-Plus spectrometer. The kinetics assays were performed using UV–Vis Lambda 25 spectrometer (Perkin-Elmer Precisely).

To study the mechanism of enzymatic oxidation of native l-DOPA and its isotopomers: [2′,5′,6′-^2^H_3_]- and [5′-^2^H]-l-DOPA the SIE and KIE methods were used. The standard mixture for kinetics experiments was consisted of 50 mM phosphate buffer, pH 7.0, or fully deuterated phosphate buffer corrected to pD 7.4, to which H_2_O_2_ and enzyme horseradish peroxidase (EC 1.11.1.7) were added to reach final concentration of 9.8 mM and 0.05 U/mL activity, respectively. Each kinetic run was consisted of six measurements carried out in 1 mL quartz spectroscopic cuvettes contained standard solution and l-DOPA or its deuterated isotopomers in concentrations in the range between 0.09 and 0.39 mM with 0.06 mM intervals. The progress of oxidation reaction was registered spectrophotometrically by measuring the increase of absorbance at *λ* = 480 nm of dopachromes formed for 20 min at 37 °C at 1-min intervals.

### Syntheses

#### Synthesis of [2′,5′,6′-^2^H_3_]-l-DOPA·DCl, **1**

For synthetic purpose 70 mg (0.355 mmol) sample of l-DOPA dissolved in 8 mL of 6 M solution DCl/D_2_O was placed in glass ampoule, frozen with liquid nitrogen, outgassed and sealed under vacuum. The exchanging mixture was kept at 50 °C for 24 h. The ampoule was broken and solvent was evaporated to about 2 mL under reduced pressure and next lyophilized. To remove deuterium from the labile positions of deuterated l-DOPA the residue was dissolved in 5 mL of water, equilibrated in room temperature for 1 h and evaporated and lyophilized to dryness. This treatment was repeated three times. As a result 84 mg (0.354 mmol) sample of **1** was obtained with 99.7 % chem. yield. The purity of the product was checked by TLC (aluminum oxide, *n*-butanol: acetic acid: water; 4:1:2; v/v/v). The extent of deuterium incorporation into the 2′, 5′ and 6′ of ring positions of **1** near to 100 % was determined by ^1^H NMR spectrum. The chemical shifts for l-DOPA; relative to TMS in D_2_O, 500 MHz: δ [ppm]: 3.026 and 3.190 (2H_β_), 3.955 (1H_α_) and 6.767, 6.856, 6.928 (3H_ring_). The signals from three aromatic ring protons of l-DOPA disappeared in **1** [16].

#### Synthesis of [5′-^2^H]-l-DOPA, **2**

##### Synthesis of deuterated [3′,5′-^2^H_2_]-l-tyrosine·DCl, **3**

To the ampoule with 208 mg (1.15 mmol) sample of l-tyrosine 1 mL conc. DCl and 5 mL D_2_O were added to reach a final conc. of DCl about 2 M. The contents of ampoule was frozen with liquid nitrogen, outgassed, sealed under vacuum and heated at 130 °C for 24 h. After this time, solvent was evaporated to about 2 mL under reduced pressure and lyophilized. To remove the deuterium from the labile position of **3** the residue was treated three times similarly as described in point 1. As a result 249 mg (1.13 mmol) sample of **3** was obtained with 98 % chem. yield. The purity of **3** was checked by TLC. The analysis of ^1^H NMR spectrum shows almost 100 % incorporation of deuterium into 3′ and 5′-ring positions of **3**. The ^1^H-chemical shifts for l-tyrosine; relative to TMS in D_2_O, 500 MHz: δ [ppm]: 3.186 and 3.306 (2H_β_), 4.313 (1H_α_), 6.927 (${\text{H}}_{{ 3^{\prime } }}$ and ${\text{H}}_{{ 5^{\prime } }}$) and 7.224 (${\text{H}}_{{ 2^{\prime } }}$ and ${\text{H}}_{{ 6^{\prime } }}$). The signals from the 3′ and 5′ protons into the aromatic ring of **3** disappeared.

##### Synthesis of [5′-^2^H]-l-DOPA, **2**

To an incubation vial, containing 25 mL of 0.1 M phosphate buffer, 32 mg (0.14 mmol) of **3** was added and acidity of the solution was adjusted to pH 6.8 using ascorbic acid. After addition of 10 kU of enzyme tyrosinase (EC 1.14.18.1) the incubation mixture was stirred for 2 h at room temperature. The reaction was quenched by removing the enzyme by centrifugation. The volume of post-reaction mixture was reduced to about 2 mL by evaporation under reduced pressure. The mixture was loaded onto a chromatographic column (200 × 10 mm) filled with aluminum oxide and unreacted **3** was washed out with 0.5 M CH_4_COONH_4_ solution. In the second step, **2** was eluted with 0.5 M CH_3_COOH. The content of each fractions was checked by TLC (silica gel, acetonitile: water; 4:1; v/v) and using color reaction of l-DOPA with KIO_3_ [17]. The fractions containing **2** were combined, concentrated under reduced pressure at room temperature and desalted onto aluminum oxide chromatographic column using water and 0.5 M CH_3_COOH. The fractions with **2** were combined again, evaporated to about 2 mL and then lyophilized. As a result 16 mg (0.08 mmol) of **2** were obtained with 57 % chem. yield. The purity of the product was checked by TLC (aluminum oxide, *n*-butanol: acetic acid: water; 4:1:2; v/v/v). The ^1^H NMR spectrum for **2** (relative to TMS in D_2_O, 500 MHz) shows disappearance of signal from 5′-ring proton (*δ* = 6.894 ppm) proving near 100 % incorporation of deuterium into the 5′ position of **2** [18].

## Results and discussion

### Syntheses

**Fig. 2:**
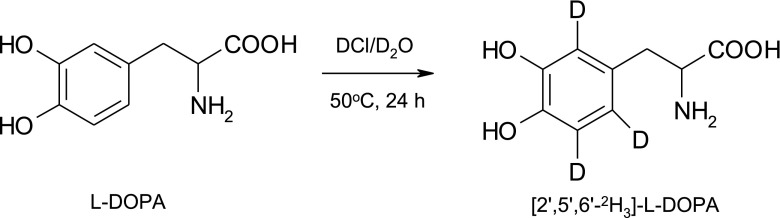
Deuterium isotopic exchange between l-DOPA and heavy water

For the synthesis of ring labeled isotopomers of l-DOPA: [2′,5′,6′-^2^H_3_]- and [5′-^2^H]-l-DOPA, needed for kinetic studies previously described methods were employed [16, 18, 19]. [2′,5′,6′-^2^H_3_]-l-DOPA was obtained using slightly modified procedure of acid-catalyzed isotopic exchange between l-DOPA and heavy water presented in Fig. 2.

The synthesis was carried out in 6 M DCl dissolved in heavy water at 50 °C for 24 h. The degree of deuterium incorporation was determined by ^1^H NMR spectroscopy. No significant change of proton NMR signal integrations corresponding to methylene and methane groups have been noticed. The incorporation of deuterium takes place only into the aromatic ring of l-DOPA and the rates of H/D exchange were practically the same for three ring protons.

**Fig. 3:**
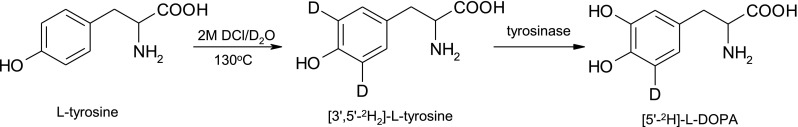
Two-step synthesis of [5′-^2^H]-l-DOPA from l-tyrosine

[5′-^2^H]-l-DOPA isotopomer was synthesized in two-step reaction, Fig. 3. In first step, the intermediate [3′,5′-^2^H_2_]-l-tyrosine was obtained by deuteriation of l-tyrosine in 2 M DCl/D_2_O at 130 °C for 24 h. Under these conditions the protons from the 3′ and 5′ of aromatic ring positions of l-tyrosine were completely substituted by a deuterium. In the second step, [5′-^2^H]-l-DOPA isotopomer was obtained using the tyrosinase-catalyzed hydroxylation of [3′,5′-^2^H_2_]-l-tyrosine. The enzyme tyrosinase from mushrooms (EC 1.14.18.1) mediates also the oxidation of l-DOPA to dopaquinone [20]. However, in the presence of ascorbic acid, this process is reversible [21]. Ascorbic acid reduces dopaquinone back to l-DOPA and becomes oxidized to dehydroascorbic acid [22]. In case of [5′-^2^H]-l-DOPA isotopomer the deuterium label into the 5′ of ring position was determined by ^1^H NMR spectroscopy.

### Kinetic assays

**Fig. 4:**
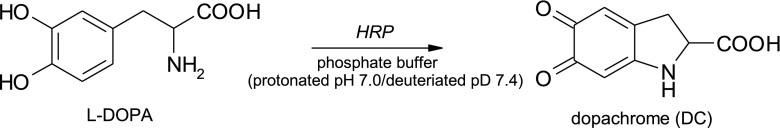
Oxidation of l-DOPA to dopachrome catalyzed by HRP

The oxidation of l-DOPA to DC, catalyzed by horseradish peroxidase, Fig. 4, was carried out in phosphate buffers (protonated or deuterated, pH 7 and pD 7.4, respectively) at 37 °C for 20 min. The kinetics has been studied spectrophotometrically by measuring the absorbance of DC at *λ* = 480 nm.

The deuterium KIEs and SIEs in the studied enzymatic oxidation of l-DOPA to DC were determined using non-competitive spectrophotometric method. The measured values of absorbance were used to calculate the reaction rates, V, and Michaelis constants, *K*_M_, using Michaelis–Menten equation [23]

1 $$ V = \frac{{V_{\hbox{max} } [S]}}{{K_{\text{M}} + [S]}}, $$

The measured kinetic parameters are optimized by Lineweaver–Burk plot (or double reciprocal plot) [24].

2 $$ \frac{1}{V} = \frac{{K_{\text{M}} + [S]}}{{V_{\hbox{max} } [S]}} = \frac{{K_{\text{M}} }}{{V_{\hbox{max} } [S]}}\frac{1}{[S]} + \frac{1}{{V_{\hbox{max} } }}, $$

where *V* is the reaction rate, *K*_M_ is the Michaelis–Menten constant, *V*_max_ is the maximum reaction rate, and [*S*] is the substrate concentration. *K*_M_ and *V*_max_ are interrelated, and they are a measure of the strength of enzyme–substrate (E–S) binding or the rate of reaction under conditions given, respectively.

The values of SIEs for l-DOPA and its deuterated isotopomers were calculated using the ratio of the *V*_max_ and *V*_max_/*K*_M_ obtained from the kinetic parameters for oxidation carried out in fully deuterated or protonated buffers. The KIEs for l-DOPA were calculated in a similar way as the SIEs from the ratio of the *V*_max_ or *V*_max_/*K*_M_ using the kinetic data obtained as a result of enzymatic oxidation of authentic and deuterated l-DOPA to DC in protonated or fully deuterated buffers. The respectively values of SIEs and KIEs for enzymatic oxidation of l-DOPA and isotopomers: [2′,5′,6′-^2^H_3_]- and [5′-^2^H]-l-DOPA are presented in the Table 1.

**Table 1:** Values of SIEs and KIEs in oxidation of l-DOPA and its deuterated isotopomers catalyzed by HRP

		l-DOPA	[2′,5′,6′-^2^H_3_]-l-DOPA	[5′-^2^H]-l-DOPA
SIE	$V_{\hbox{max} } /(V_{\hbox{max} } )^{\text{a}}$	2.1 ± 0.1	2.4 ± 0.2	2.1 ± 0.16
	$\frac{{V_{\hbox{max} } /K_{M} }}{{(V_{\hbox{max} } /K_{M} )^{\text{a}} }}$	1.32 ± 0.07	1.61 ± 0.03	1.1 ± 0.06
KIE (H_2_O)	$V_{\hbox{max} } /(V_{\hbox{max} } )^{\text{a}}$	–	1.1 ± 0.1	2.2 ± 0.14
	$\frac{{V_{\hbox{max} } /K_{M} }}{{(V_{\hbox{max} } /K_{M} )^{\text{a}} }}$	–	1.72 ± 0.04	3.2 ± 0.09
KIE (D_2_O)	$V_{\hbox{max} } /(V_{\hbox{max} } )^{\text{a}}$	–	1.29 ± 0.08	2.2 ± 0.2
	$\frac{{V_{\hbox{max} } /K_{M} }}{{(V_{\hbox{max} } /K_{M} )^{\text{a}} }}$	–	2.1 ± 0.1	2.7 ± 0.2

^a^Means values for deuterated compounds

The developed method allowed to determine for the first time the deuterium SIEs and KIEs in the oxidation of l-DOPA to DC catalyzed by HRP and provide new information about the mechanism of this reactions.

As can be see from Table 1, the oxidation of l-DOPA in protonated buffer proceeds about two times faster than in fully deuterated buffer (SIE on *V*_max_ = 2.1–2.4). This indicates that formation or breaking of C-D bond needs higher energy than in case of C–H bond. The values of SIEs for the oxidation of native l-DOPA and its two deuterated isotopomers are similar in each studied case and typical for secondary isotope effects, whereas the small values show that the active complex is only slightly stabilized by interactions with solvent-substrate molecules. The values of SIEs on *V*_max_ = 2.1 and 2.4 suggest that solvent has a little effect on the conversion of “E–S” complex to “enzyme–product” (E–P) complex. On the other hand, even lower values of SIEs on *V*_max_/*K*_M_ indicate that the solvent has a negligible role during formation of E–S complex [15].

The values of deuterium KIEs on *V*_max_ and *V*_max_/*K*_M_ in the studied reaction for [5′-^2^H]-l-DOPA are characteristic of secondary isotope effect and suggest that the presence of deuterium atom in this position probably has a little impact on the course of oxidation reaction. The values of KIE on *V*_max_ = 2.2 indicate that the deuterium substitution slightly affects the conversion of E–S complex to E–P complex [25]. Moreover, a little higher value of KIE on *V*_max_/*K*_M_ = 3.2 and 2.7 (in protonated and deuterated buffers, respectively) in this case suggests that the hydrogen atom at the 5′ of ring position plays the primary role in the binding of the E–S. It is quite possible that the secondary isotope effects in studied oxidation reaction may appear due to hybridization or hyperkonjugation, occurring during the formation of the active complex in step determining the reaction rate [26].

The values of deuterium KIEs on *V*_max_ and *V*_max_/*K*_M_ close to unity (no isotope effect) for second studied isotopomer: [2′,5′,6′-^2^H_3_]-l-DOPA seem to be typical for secondary isotope effects. Undoubtedly, a substitution in 5′ of ring position would confirm this assumption. However, the non-selective labeling in this isotopomer causes that the calculated values may be the result of a three effects overlapping: primary, secondary and no isotope effect (substitution at 6′, 5′ and 2′ of ring position, respectively).
